# Utility of home doppler blood pressure measurement to minimise unnecessary investigations in children with suspected hypertension

**DOI:** 10.1038/s41371-025-01038-0

**Published:** 2025-07-09

**Authors:** Manson Chon In Kuok, Joanna Newton, Cheentan Singh, Manish D. Sinha

**Affiliations:** 1 Department of Paediatric Nephrology, Evelina London Children’s Hospital, London, UK; 2 North Middlesex University Hospital, London, UK; 3 Kings College London, London, UK

**Keywords:** Diagnosis, Hypertension

## Abstract

An accurate measurement of blood pressure (BP) is essential in making a diagnosis of hypertension, however lack of reference values in children <5 years old makes out-of-office BP measurement using ambulatory blood pressure monitoring difficult in this age group. We conducted a retrospective analysis in children aged under 5 years referred to our hypertension service for suspected hypertension. We included those who underwent home doppler blood pressure measurement (HDBPM) for hypertension diagnosis confirmation, and evaluated the investigations performed for suspected hypertension before the diagnosis was confirmed using out-of-office  BP measurements. Children receiving anti-hypertensive medication at the time of initial review were excluded. Fifty-five children (62% male) with a median age of 1.6 years completed HDBPM and were included. Nearly 90% of them referred for hypertension were found to be normotensive following out-of-office BP assessment using HDBPM. In these normotensive patients, different investigations for secondary hypertension, including blood tests (creatinine, renin, aldosterone, cortisol, thyroid function tests, and catecholamine levels), doppler kidney ultrasound, and echocardiograms were performed before referral, most of which yielded unremarkable results. Our finding suggested that whilst some initial investigations were essential, second-line tests for less common secondary causes of hypertension are often unnecessary before hypertension is confirmed. We recommend deferring some of these investigations in asymptomatic children until hypertension is verified by home blood pressure measurements.

## Introduction

Use of out-of-office measurement for diagnosing and/or ruling out hypertension can potentially reduce unnecessary investigations and hospitalisations related to elevated blood pressure in office settings. An accurate assessment of blood pressure (BP) is critical when diagnosing hypertension, and out-of-office measurements help identify white-coat and masked hypertension. Ambulatory BP monitoring (ABPM) is a reliable and reproducible method for diagnosing hypertension in children and adolescents [1]. However, ABPM is less likely to be tolerated by younger patients, and the lack of normative data for children under 5 years old prevents accurate interpretation in this age group [2].

We have previously shown home doppler blood pressure measurement (HDBPM) to be a satisfactory, reliable and acceptable method to parents [3–5]. It offers a practical way of monitoring BP at home in the younger age group.

In this study, we describe the characteristics of children under 5 years old who underwent HDBPM for hypertension diagnosis confirmation, and to evaluate the results of investigations performed, to identify suspected secondary hypertension prior to confirmation of hypertension using out-of-office measurements.

## Methods

We retrospectively reviewed all <5 years old patients who were referred to our quaternary paediatric hypertension service at Evelina London Children’s Hospital, UK between 2017 and 2021. We included all patients who were referred with a clinical suspicion of hypertension, to confirm diagnosis and who underwent out-of-office BP measurements using HDBPM. Those already receiving anti-hypertensive medications or unable to complete HDBPM were excluded from the analysis.

HDBPM was indicated to parents if (i) they felt comfortable with home BP measurement (ii) they could understand instructions in English, and (iii) at least two adult caregivers were available for those <2 years old as additional challenges in BP measurement were anticipated in this age group. The method of HDBPM, arrangement of educational sessions and phone follow-up by the hypertension clinical nurse specialists have been previously reported [5]. Hypertension was defined using SBP > 95th percentile value for respective sex, age and height as per the 2016 European Society of Hypertension guidelines for those aged 1–17 years [6], and the same percentile cut-off in Second Task Force normative values for those <1 year old [7].

Demographic data, referral details, clinical assessments and HDBPM results were retrieved from the electronic health records. To minimise missing data, relevant details if not available in the records, were collated by contacting the referring hospitals. Children initially assessed for unrelated illnesses who were found to have elevated BP were classified into ‘incidental group’, while those with underlying conditions that posed a higher risk of hypertension were put in the ‘surveillance group’.

We identified whether patients had chronic kidney disease (CKD) and if CKD in them was due to congenital anomalies of the kidney and urinary tract (CAKUT) or any other underlying aetiologies. Perinatal history was considered to be abnormal if there was reported admission to neonatal intensive care unit.

Data are presented as medians and interquartile ranges (IQR) when not normally distributed and compared between groups using Mann-Whitney U tests. Categorical data were expressed as frequencies and percentages and analysed by Pearson chi-square tests or Fisher exact tests as appropriate. Statistical analyses were performed using IBM Statistical Package for Social Sciences (SPSS) for Windows Version 26.0. A *p*-value of less than 0.05 was considered statistically significant.

## Results

This analysis included 55 patients (34 boys, 62%), who had a median (IQR) age of 1.6 (0.8, 2.9) years old at referral. There were 29 (53%) patients in the incidental group, and the rest were identified during BP surveillance for underlying conditions (surveillance group). There were 29 patients (53%) with CKD. Of these, 26 (90%) had CKD due to CAKUT, while the remaining 3 (10%) had CKD following perinatal hypoxia. The majority of CKD patients (88%, 23/29) were in the surveillance group.

The median (IQR) of systolic blood pressure (SBP) and corresponding SBP z-score at the time of referral were 119 (112, 126) mmHg and 2.77 (1.78, 3.30), respectively. Sixteen (29%) patients were acutely unwell when elevated BP was first noted, and they had a higher referral SBP (125 vs. 115 mmHg, *p* = 0.008) and SBP z-score (3.22 vs. 2.55, *p* = 0.016). Four patients were referred based solely on BP readings taken from the lower limbs. The demographics and clinical characteristics between the incidental and surveillance groups are compared in Table 1.

**Table 1:** Demographics and clinical characteristics of incidental group and surveillance group.

Variables	All patients (*n* = 55)	Incidental group (*n* = 29)	Surveillance group (*n* = 26)	*p*-value
Gender (Male)	34 (62%)	19 (66%)	15 (58%)	0.551
Age at referral (Years)	1.6 (0.8, 2.9)	1.6 (0.9, 2.8)	1.5 (0.8, 3.7)	0.781
Chronic kidney diseases	29 (53%)	6 (21%)	23 (88%)	**<0.001**
Family history of hypertension	7 (13%)	5 (17%)	2 (8%)	0.426
Referral source				
Primary	1 (2%)	1 (3%)	0	**0.032**
Secondary	28 (51%)	19 (66%)	9 (35%)	
Tertiary	26 (47%)	9 (31%)	17 (65%)	
SBP at referral (mmHg)	119 (112, 126)	120 (115, 129)	115 (105, 122)	**0.023**
SBP (z-score) at referral	2.77 (1.78, 3.30)	2.81 (2.33, 3.64)	2.01 (1.48, 3.20)	0.118
High BP detected when acutely ill	16 (29%)	16 (55%)	0	**<0.001**
Confirmed hypertension^a^	7 (13%)	2 (7%)	5 (19%)	0.171
Confirmed normotension^a^	48 (87%)	27 (93%)	21 (81%)	

Data are shown as N (percentage) or median (interquartile ranges).

Bold values indicate statistical significance *p* < 0.05.

^a^Diagnosis was confirmed following out-of-office blood pressure measurements using HDBPM.

**Fig. 1:**
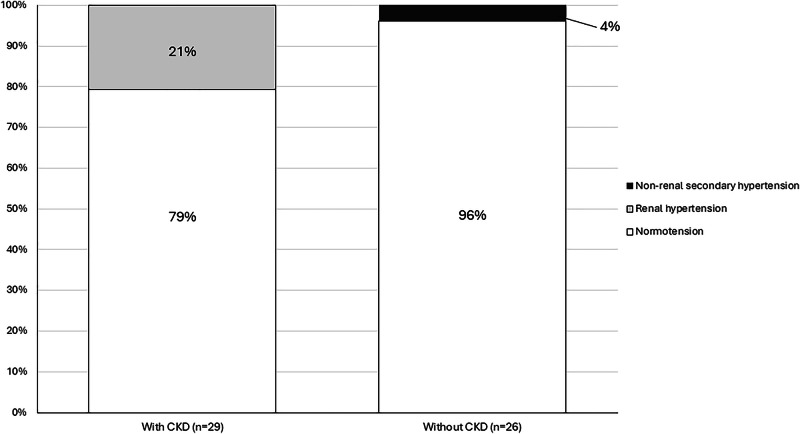
Diagnosis of hypertension following home doppler blood pressure monitoring in patients with and without CKD.

Following HDBPM, a diagnosis of hypertension was confirmed only in 7 (13%) children, all of whom had secondary hypertension. Six had CKD-related hypertension and one had suspected adrenal pathology (Fig. 1). Abnormal perinatal history was significantly associated with confirmation of hypertension (86 vs. 17%, *p* = 0.001). Logistic regression analysis did not identify any additional risk factors — including sex, age, growth parameters (height, weight, BMI, and their respective z-scores), referral systolic blood pressure (SBP), or SBP z-score — as significant predictors of hypertension.

Among patients confirmed to be normotensive following HDBPM (*n* = 48), there was marked variability in the investigations and monitoring of patients prior to referral (Table 2). Overnight hospitalisation for BP monitoring was arranged in 5 (10%) patients. Serum creatinine (43/48, 90%) and kidney ultrasound (USS) (40/48, 83%) were the most common investigations performed prior to HDBPM.

**Table 2:** Summaries of investigations and results in patients confirmed as normotensive following out-of-office blood pressure measurements using HDBPM (*n* = 48).

	Incidental group (*n* = 27)		Surveillance group (*n* = 21)	
	Investigations done	New abnormal findings	Investigations done	New abnormal findings
Blood tests				
Creatinine	25 (93%)	0	18 (86%)	1 (6%)
Renin	5 (19%)	0	0	–
Aldosterone	5 (19%)	0	0	–
Lipid profile	4 (15%)	1 (25%)	0	–
Thyroid function	7 (26%)	0	0	–
Cortisol	5 (19%)	0	0	–
Plasma catecholamine	2 (7%)	0	0	–
Urine tests				
Urine ACR/PCR	12 (44%)	4 (33%)	8 (38%)	1 (13%)
Urine catecholamine	2 (7%)	0	0	–
Imaging				
USS Kidney	22 (81%)	4 (18%)	18 (86%)	0
USS Doppler	14 (52%)	0	1 (5%)	0
Echocardiogram	13 (48%)	0	6 (29%)	0

*ACR* albumin-creatinine ratio, *PCR* protein-creatinine ratio, *USS* ultrasound.

In those with confirmed normotension and categorised in the study incidental group (*n* = 27), urine samples were collected for albumin-creatinine ratio or protein-creatinine ratio in 44% (12/27), with one-third showing mild elevation. Around half of patients in the incidental group had undergone renal Doppler ultrasound (52%, 14/27) and echocardiogram (48%, 13/27). Only 18% (4/27) had abnormal findings on kidney ultrasound (nephrocalcinosis, hydronephrosis, duplex kidney, and increased cortical echogenicity). Other blood tests—including thyroid function tests, serum cortisol, renin and aldosterone levels, and plasma and urine catecholamine levels—were performed in fewer than 25% of patients, none with any abnormal findings (Table 2). In those with confirmed normotension and categorised in the study in the surveillance group, the yield for new abnormal findings for the investigations was similarly low, with no new findings obtained from renal or cardiac imaging.

## Discussions

In this report, we observed that a significant portion of patients under 5 years old who were referred for suspected secondary hypertension were in fact normotensive following out-of-office BP measurements using HDBPM, and the results of the initial investigations had a very low yield of clinically relevant findings.

In young children aged <6 years old, secondary hypertension remains the most prevalent cause of hypertension [8, 9]. Over the past decade, several guidelines on paediatric hypertension have been published, all recommending that obtaining reliable and accurate BP measurements is essential. Following confirmation of hypertension, investigations to identify secondary causes of hypertension, associated co-morbidities, and potential end-organ damage should be performed [10].

Out-of-office BP monitoring is recommended in the diagnostic pathway of hypertension, as it can identify white coat hypertension, and has better reproducibility than office measurements [11, 12]. ABPM is the standard method for obtaining multiple out-of-office measurements for diagnosis, but it is not suitable for children younger than 5 years old due to a lack of normative data [13]. Home BP monitoring with oscillometric devices is recommended in older children and adolescents, but its use is limited in young infants due to concerns regarding accuracy [14]. HDBPM provides an alternative method for obtaining systolic BP measurements at home in this age group. In our experience, HDBPM by parents in this age group has been well-received, with high parental compliance, reliability and success rate [4, 5].

Out-of-office BP monitoring also plays an important role in ruling out hypertension, thereby reducing unnecessary investigations and associated costs [15]. In young patients referred for hypertension but subsequently found to be normotensive following HDBPM, our analysis showed that most investigations yielded no abnormal results. These findings suggest that such investigations are unnecessary unless hypertension is confirmed through out-of-office measurements. Some patients were referred due to elevated BP readings during acute illness or post-operative periods, when BP is typically higher than the baseline values. Besides, some referrals were based on lower limb measurements, which can be falsely elevated and may not accurately correlate with upper limb measurements [16–18]. These seemingly elevated BP readings have led to unnecessary investigations and admissions.

In the surveillance group, nearly 90% of the patients had known CKD, and have a substantially elevated risk of early-onset hypertension. Of note, our cohort demonstrated a strong association between abnormal perinatal history and hypertension, likely due to factors inherent with kidney diseases including immature nephrogenesis and vascular dysfunction in prematurity, and increased susceptibility to kidney insults from various perinatal complications [19]. This highlights the importance of a low threshold for arranging out-of-office BP monitoring in this group of patients to facilitate early detection and interventions, thereby mitigating further kidney damage.

We recognise there may be a time lag between the initial suspicion of hypertension and confirmation. We recommend that some preliminary investigations, such as serum creatinine, urinalysis and USS kidney, be considered at referral, as renal abnormalities are common contributors to secondary hypertension in young children, especially those with a history of urinary tract infection or renal insults [9, 20]. Additional investigations, such as echocardiogram, tests for renin and aldosterone, cortisol, thyroid function and catecholamines, should be performed once hypertension is confirmed. Exceptions should be made if there are concurrent signs and symptoms suggestive of secondary causes, such as thyrotoxicosis, coarctation of aorta, the presence of an abdominal mass or bruits above the renal arteries. This pragmatic approach would facilitate the early diagnosis or exclusion of common renal pathologies in patients with suspected hypertension, while also reducing unnecessary investigations and associated costs.

There are several limitations related to our study findings. Firstly, owing to its retrospective nature, there could be missing data regarding the investigations conducted in other centres. Details regarding the BP measurement methods and their sites were often not documented despite attempts to complete missing information by contacting the referring hospitals. Secondly, HDBPM was not able to detect isolated diastolic hypertension. All patients had their blood pressure initially checked in clinic with no patients showing isolated diastolic hypertension [21]. Thirdly, we did not systematically evaluate the characteristics of patients who did not undergo HDBPM. Fourthly, we accept that the relatively small sample size in this single centre review may limit the generalisability of our study findings. Lastly, we accept that HDBPM, although valuable for confirming hypertension, may not be readily available in all centres treating young children.

Nonetheless, our findings provide insights into the role of out-of-office BP monitoring in excluding hypertension in young children, therefore avoiding unnecessary investigations and hospitalisations. This approach can reduce parental anxiety, and minimise unnecessary healthcare burden.

## Conclusion

Our study showed that nearly 90% of patients <5 years old referred for hypertension were normotensive following out-of-office measurement with HDBPM. Second-line investigations can potentially be deferred until hypertension is confirmed by home BP measurements. For patients with known renal condition, HDBPM would aid early diagnosis of evolving hypertension and its management. Should patients present with clear clinical signs indicating an underlying cause of hypertension, such as thyrotoxicosis or an abdominal mass, urgent investigations are warranted. However, since the majority of secondary hypertension in childhood is renal-related, a pragmatic approach would involve initiating standard investigations, such as serum creatinine, urine tests for proteinuria, and renal ultrasound to help bridge the time gap before referral for out of office blood pressure monitoring in young children.

## Summary table

### What is known about the topic

- Out-of-office blood pressure measurement plays a crucial role in both diagnosing and ruling out hypertension.
- Ambulatory blood pressure monitoring in children under 5 years old is difficult due to poor tolerance and lack of normative data for interpretation.

### What this study adds

- Nearly 90% of children under 5 years old referred for suspected hypertension were found to be normotensive with home doppler blood pressure measurement (HDBPM).
- Different investigations, including blood tests and imaging, were performed before out-of-office measurements were conducted, and most of these results did not show any abnormality.

## Data Availability

Data are available from authors upon reasonable request.
